# Delivering two new treatments for malaria: a story of inventive partnerships

**DOI:** 10.1186/1475-2875-11-S1-P57

**Published:** 2012-10-15

**Authors:** Graciela Diap, Piero Olliaro, Jean-René Kiechel

**Affiliations:** 1 Drugs for Neglected Diseases initiative, 1202 Geneva,, Switzerland; 2 UNICEF/ UNDP/World Bank/WHO Special Programme for Research and Training in Tropical Diseases (TDR), 1211 Geneva 27, Switzerland

## 

The World Health Organisation (WHO) currently recommends the use of five artemisinin-based combination therapies (ACTs) for the treatment of uncomplicated *Plasmodium falciparum* malaria. ACT combines two effective antimalarial drugs and is the cornerstone of successful malaria control and ultimately, elimination. Fixed-dose combinations (FDC) of ACTs are preferred, as they promote adherence to treatment and reduce the risk of selecting for drug-resistant parasites.

In order to strengthen the ACT portfolio of FDCs, which at the time was minimal, the Drugs for Neglected Diseases *initiative* (DND*i*), together with the WHO Special Programme for Research and Training in Tropical Diseases (TDR), launched the FACT (Fixed Dose Artesunate Combination Therapy) project in 2002, with original funding from INCO/DEV. The FACT core group also included the Brazilian government-owned pharmaceutical company, Farmanguinhos/Fiocruz, the University of Bordeaux, Universiti Sains Malaysia, Mahidol University and the Shoklo Malaria Research Unit in Thailand, the Centre National de Recherche et de Formation sur le Paludisme in Burkina Faso and the University of Oxford, combining epidemiological and drug development expertise. Additional competencies in clinical studies, clinical supplies and regulatory matters strengthened the core team. Through innovative collaborations with this variety of partners, the FACT project delivered FDCs of Artesunate (AS) plus Amodiaquine (ASAQ), and AS plus Mefloquine (MQ). Key contributions in scale-up, industrial production and regulatory filing were still needed to make the products available to patients, and different strategies were set up for each product. Both strategies were based on an initial development within the public and not-for-profit sector, with an extension to the private sector when a viable product was available, for registration, production and distribution.

Sanofi took over the last steps of industrial development and regulatory filing for ASAQ and the product was pre-qualified by WHO in 2008. Since then, ASAQ has been registered in over 30 countries, mostly in sub-Saharan Africa and over 120 million treatments have been delivered - making this the second most widely used ACT. Deployment was accompanied by a risk-management plan, together with an extensive training and educational programme developed by Sanofi. ASMQ FDC production was scaled up by Farmaguinhos/Fiocruz and registered in Brazil in 2008. A large intervention study involving over 23,000 patients was performed by the National Malaria Control Programme in Brazil showing the efficacy of the product in real-life conditions. Through a South-South technology transfer, ASMQ FDC production was transferred to the Indian pharmaceutical company Cipla to ensure availability in India and South East Asia. ASMQ FDC was registered in India in 2011 and in Malaysia in early 2012 and is under review for WHO prequalification.

The FACT project demonstrates that alternative drug development strategies can make safe, affordable and sustainable treatments available to patients. Diverse partnerships gathering a wide range of expertise, from national programmes ensuring the most appropriate therapy is used in endemic areas to industrial partners for product implementation and wide distribution, were key to success. Both ASAQ and ASMQ FDCs are critical drugs for malaria control across Africa, Latin America and South East Asia.

